# Preparation of Electrochemical Sensor Based on Zinc Oxide Nanoparticles for Simultaneous Determination of AA, DA, and UA

**DOI:** 10.3389/fchem.2020.592538

**Published:** 2020-11-25

**Authors:** Yuanzhi Pan, Junli Zuo, Zhongyu Hou, Yizhong Huang, Cancan Huang

**Affiliations:** ^1^National Key Laboratory of Science and Technology on Micro/Nano Fabrication, Key Laboratory for Thin Film and Microfabrication of Ministry of Education, Department of Micro/Nano Electronics, School of Electronic Information and Electrical Engineering, Shanghai Jiao Tong University, Shanghai, China; ^2^Central Academe, Shanghai Electric Group Co., Ltd., Shanghai, China; ^3^Zhenjiang Hongxiang Automation Technology Co., Ltd., Zhenjiang, China; ^4^Department of Geriatrics, Ruijin Hospital, Shanghai Jiao Tong School of Medicine, Shanghai, China; ^5^School of Materials Science and Engineering, Nanyang Technological University, Singapore, Singapore; ^6^Beijing Kanghong Biomedical Co., Ltd., Beijing, China

**Keywords:** electrochemical sensor, zinc oxide, ascorbic acid, dopamine, uric acid

## Abstract

ZnO nanoparticles (NPs) were synthesized using a hydrothermal method. Scanning electron microscope (SEM) and X-ray diffraction have been used for characterizing the synthesized ZnO NPs. An electrochemical sensor was fabricated using ZnO NPs–modified glassy carbon electrode for simultaneous determination of ascorbic acid (AA), dopamine (DA), and uric acid (UA). The proposed electrochemical sensor exhibited excellent detection performance toward three analytes, demonstrating that it can potentially be applied in clinical applications. The results indicated the ZnO NPs–modified electrode can detect AA in the concentrations range between 50 and 1,000 μM. The ZnO NPs–modified electrode can detect DA in the concentrations range between 2 and 150 μM. The ZnO NPs–modified electrode can detect UA in the concentrations range between 0.2 and 150 μM. The limits of detections of AA, DA, and UA using ZnO NPs–modified electrode were calculated to be 18.4, 0.75, and 0.11 μM, respectively.

## Introduction

Ascorbic acid (AA), dopamine (DA), and uric acid (UA) are active substances with important biological research value existing in the extracellular fluid of the human central nervous system. Among them, AA plays an important role in promoting the growth of organisms and synthesizing antibodies (Ejaz and Jeon, 2017; Fu et al., 2018). As an important biological small molecule substance in the human central nervous system, the content of DA in the body below or beyond the normal level will directly affect the mental activities of the human body (Atta et al., 2019). When purine metabolism is abnormal in the human body, excessive UA can be produced; UA retained in the body will change the pH value of body fluid and form an acidic internal environment, which has an important impact on the function of somatic cells (Hsu et al., 2017; Nagles et al., 2017). In view of the important medical research value of detecting the contents of these three substances, rapid and accurate detection methods are essential for the diagnosis (Abellán-Llobregat et al., 2018). In recent years, the detection of AA, DA, and UA has attracted considerable attention (Fu et al., 2019, 2020; Shamsadin-Azad et al., 2019; Karimi-Maleh et al., 2020c; Zhou et al., 2020).

At present, the common detection methods of AA are spectrophotometry, chromatography, fluorescence, and electrochemical sensor (Gopalakrishnan et al., 2018; Atta et al., 2020). The principle of spectrophotometry is that AA reacts with reagent to form chromogenic group and deoxyascorbic acid through redox or derivatization reaction with reagent, thus indirectly realizing the detection of AA concentration. This method is simple and has good selectivity, but the dyes involved in the reaction are unstable and easily interfered by sulfhydryl, reducing ketone and sulfite plasma. High-performance thin-layer chromatography (HPLC) uses silica particles with narrow particle size as adsorbent, which has obvious advantages in the separation effect. Gas chromatography has good selectivity in the determination of trace substances (Zhang et al., 2018a,b). However, AA is a polar compound, which requires a series of sample pretreatment and increases the complexity of determination. The fluorescence method is a direct or indirect detection method based on the fluorescence intensity quenching and recovery of the probe after adding AA. Fluorescence detection of AA has strong interference ability and high sensitivity, which is suitable for the rapid detection of trace AA in actual samples (Zhang et al., 2018c; de Faria et al., 2020). The detection methods of DA include chemiluminescence, spectrophotometry, fluorescence, liquid chromatography, and electrochemical sensors. The detection principle of chemiluminescence is that the chemical energy absorbed by the material is converted into light energy when the chemical reaction occurs (Cinti et al., 2018). The content of the material in the sample is reflected by the luminous intensity. In spectrophotometry, the complex is formed by the reaction between DA and chromogenic agent. The absorbance of the complex at a specific absorption wavelength has a certain linear relationship with the concentration of DA. The fluorescence method can detect the content of DA in pharmaceuticals by measuring the fluorescence intensity (Ghanbari and Hajian, 2017; Long and Fu, 2017). Compared with other methods, HPLC has a higher separation rate. Because the DA itself has fluorescence, the combination of fluorescence and HPLC as an effective detection method has been widely concerned in the analysis of DA. At present, phosphotungstic acid reduction (PAR) method, HPLC, enzyme method, and electrochemical sensor have been established in clinical setting to detect UA. The principle of determination of UA by PAR is that under alkaline conditions, phosphotungstic acid reacts with UA to produce tungsten blue and allantoin (Feng et al., 2020; Hou et al., 2020; Karimi-Maleh et al., 2020a). The concentration of UA is indirectly obtained by colorimetry. This method has good accuracy for the detection of UA, but it requires higher purity of phosphotungstic acid. HPLC has the advantages of simple mobile phase and good separation effect, but the complex pretreatment of samples is time-consuming. The enzyme detection of UA is to use enzyme to catalyze the decomposition of UA to get a certain concentration of product and then calculate the content of UA (Karimi-Maleh and Arotiba, 2020; Karimi-Maleh et al., 2020a). The enzyme method for UA detection is relatively simple; however, the high cost of enzyme and the constant temperature of the reaction process limit its application.

The specificity of recognition between enzyme and substrate makes the enzyme sensor have high selectivity and low detection limit. However, the activity of the enzyme is greatly affected by external environmental factors such as pH, temperature, and material toxicity, which makes the enzyme sensor have low stability and short life. At the same time, because of the limited types of enzymes, its application scope is greatly limited. In order to overcome the shortcomings of enzyme sensors, many scientists have developed a series of enzyme-free sensors with good stability, simple preparation, and low cost. Nanomaterials have the advantages of large specific surface area, many surface-active sites, high conductivity, good adsorption performance, and strong catalytic performance, which can greatly improve the sensitivity and stability of the sensor. They can be used to immobilize biomolecules and as biomarkers to label biomolecules. They can be used as catalysts in electrochemical reactions to catalyze reactions and enhance the efficiency of electron transfer. In this work, we report the preparation of ZnO nanoparticles (NPs) using a simple one-pot synthesis method (Yumak et al., 2011; Naderi Asrami et al., 2020). The formed ZnO NPs have been used for glassy carbon electrode (GCE) modification and used as a sensitive electrochemical sensor for simultaneous detection of AA, DA, and UA. As oxidation potentials of AA, DA, and UA severely overlap, their electrochemically simultaneous determination is still a challenge. The modification of ZnO could successfully separate three analyte oxidation peaks.

## Materials and Methods

Zinc nitrate hexahydrate [Zn(NO_3_)_2_·6H_2_O], UA, DA, AA, and hydrazine were purchased from Yeyuan Biotech. Co. Ltd. Phosphate-buffered solution was prepared by mixing K_2_HPO_4_ and KH_2_PO_4_ to appropriate 0.1 M with an appropriate pH. All reagents were of analytical grade. Millipore Milli-Q water (18 MΩ cm) was used throughout.

The preparation of ZnO NPs has been carried out by previous report (Fu and Fu, 2015). Briefly, 20 mL of zinc nitrate solution (0.05 M) was prepared under stirring. Then 0.5 mL hydrazine solution (1 wt %) was added into the solution. The slurry was sonicated for 0.5 h and transferred into a Teflon-lined stainless-steel autoclave. The autoclave was heated to 120°C for 2 h. The precipitate (denoted as ZnO NPs) was collected after centrifugation and dried in an oven.

All electrochemical measurements were performed using CHI 760 electrochemical workstation with a conventional three-electrode system. A platinum wire, a 3 M Ag/AgCl electrode, and a GCE were as auxiliary electrode, reference, and working electrode, respectively. The GCE was modified by the ZnO NPs dispersion and coated with a layer of Nafion before analysis.

## Results and Discussion

**Figure 1:**
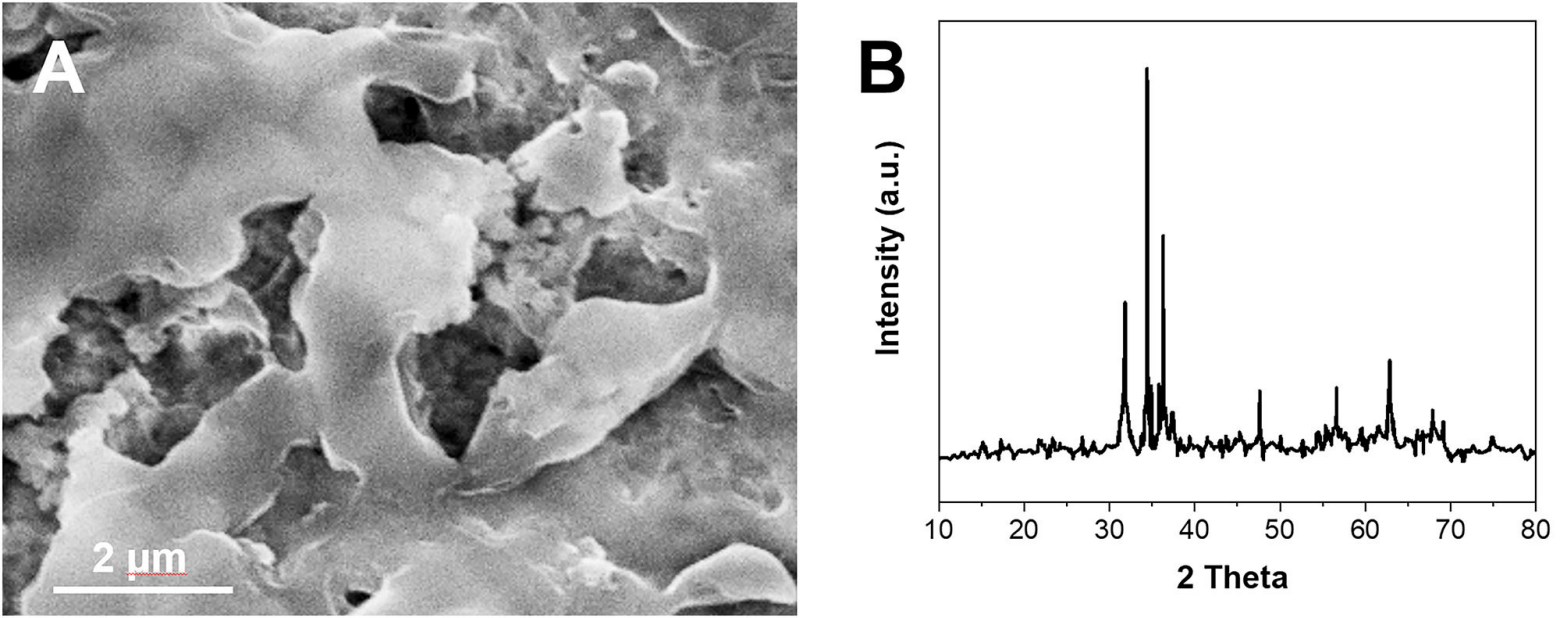
**(A)** SEM image and **(B)** XRD pattern of synthesized ZnO NPs.

Figure 1A shows the scanning electron microscope image of ZnO NPs after coating with Nafion film. It can be seen that a thin layer of the Nafion was covered above the ZnO NPs, which could prevent the detachment of the NPs during the electrochemical reaction. The formation of ZnO NPs was investigated by X-ray diffraction (XRD) (Figure 1B). The XRD pattern of the ZnO NPs displays the peaks at 31.5°, 34.4°, 36.4°, 47.2°, 56.1°, 62.8°, and 68.2°. These peaks can be indexed to hexagonal wurtzite ZnO (JCPDS 36-1451). The average size of the ZnO NPs can be calculated to 32.3 nm using the Debye–Scherrer equation.

**Figure 2:**
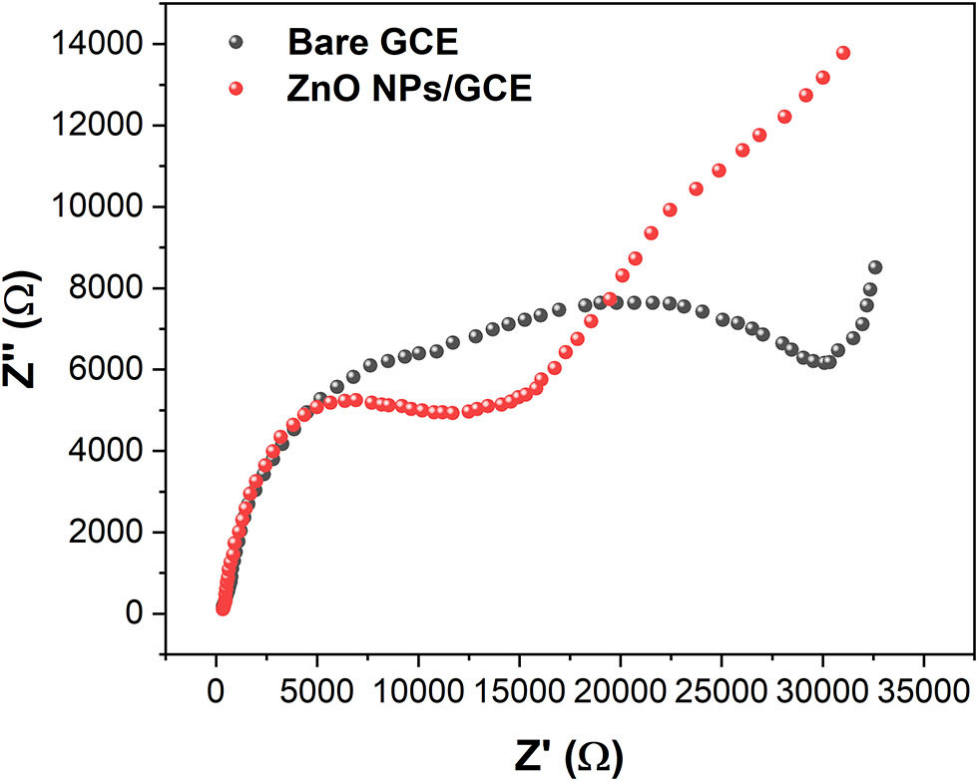
EIS plots of bare GCE and ZnO NPs/GCE.

The electrochemical property of the synthesized ZnO NPs–modified GCE and bare GCE were investigated by electrochemical impedance spectroscopy (EIS). The EIS plot of bare GCE exhibited a larger semicircle compared with that of the ZnO NPs/GCE (Figure 2), suggesting the modification of ZnO NPs could lower the electron-transfer resistance of GCE. This result indicates the modification of ZnO NPs can enhance the electrochemical property of the GCE. Therefore, it is expected to have a higher electrochemical response when interacting with analytes (Karimi-Maleh et al., 2020b).

**Figure 3:**
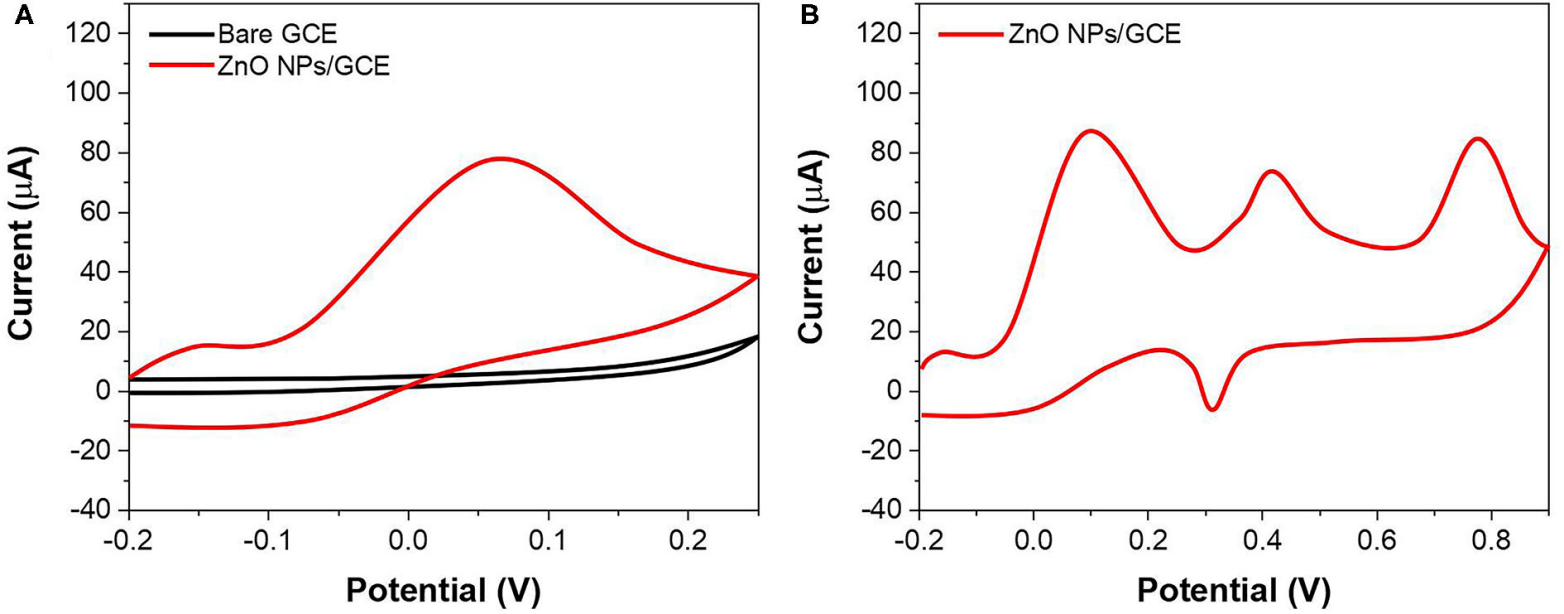
**(A)** CV of bare GCE and ZnO NPs/GCE toward 500 μM AA. **(B)** CV of ZnO NPs/GCE at mixture of AA, DA, and UA.

The electrocatalytic activity of the ZnO NPs was studied using AA as an analyte and shown in Figure 3A. It can be seen that the CV of bare GCE exhibited no distinct response toward 0.5 mM AA oxidation, whereas the ZnO NPs/GCE showed a clear oxidation peak of AA at 0.07 V. The superior sensing activity can be ascribed to the enhanced conductivity by ZnO NPs with the intrinsic electrocatalytic property. In addition, the ZnO NPs–modified GCE showed a larger background compared with that of the bare GCE, suggesting the ZnO NPs increase the electroactive surface area of the electrode. Then, the electrocatalytic behavior of the ZnO NPs/GCE was further studied using all three analytes. Figure 3B shows the CV profiles of ZnO NPs/GCE toward AA, DA, and UA. Three well-separated oxidation peaks were noticed at 0.08, 0.42, and 0.79 V, corresponding to the oxidation of UA, DA, and AA, respectively. This observation indicates the prepared ZnO NPs/GCE can be used as an electrochemical sensor for AA, DA, and UA simultaneous detection.

**Figure 4:**
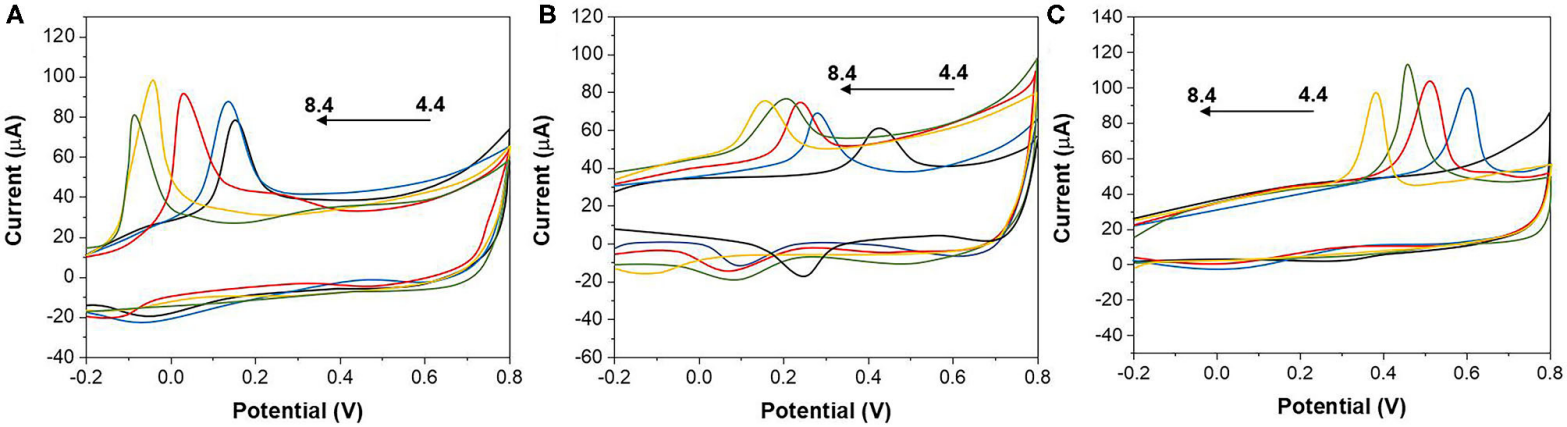
CVs of **(A)** 1,000 μM AA, **(B)** 100 μM DA, and **(C)** 100 μM UA at the ZnO NPs/GCE values of 4.4, 5.4, 6.4, 7.4, and 8.4.

The effect of pH on the detection of AA, DA, and UA using ZnO NPs/GCE was investigated. Figures 4A–C show the CV profiles of ZnO NPs/GCE toward AA, DA, and UA in the range of 4.4–8.4, respectively. The increasing of peak current was observed in all three cases when the pH increases until 7.4. Then, further increasing of pH leads to the decreasing of the current response. Therefore, pH 7.4 was selected to be an optimum condition.

**Figure 5:**
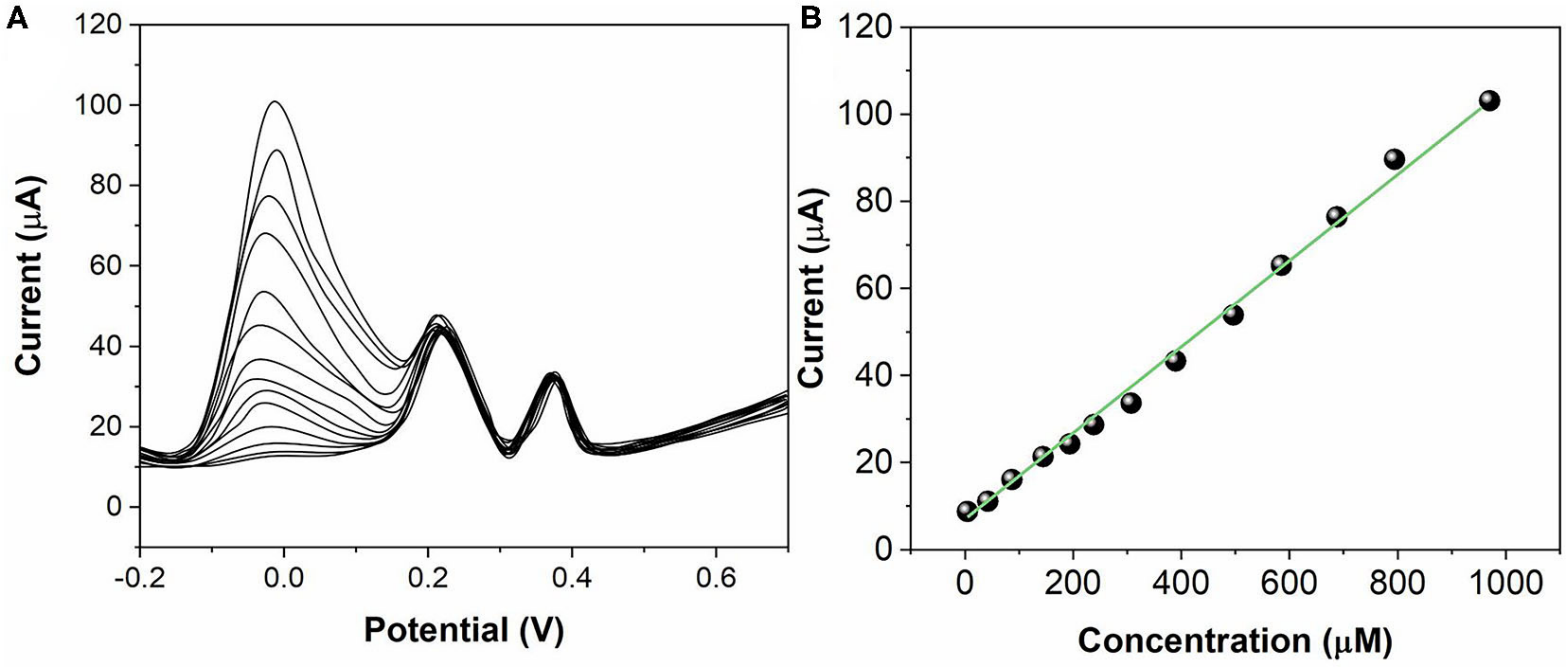
**(A)** SWVs for AA (50–1,000 μM) at ZnO NPs/GCE. **(B)** Calibration plots of AA concentration vs. current.

**Figure 6:**
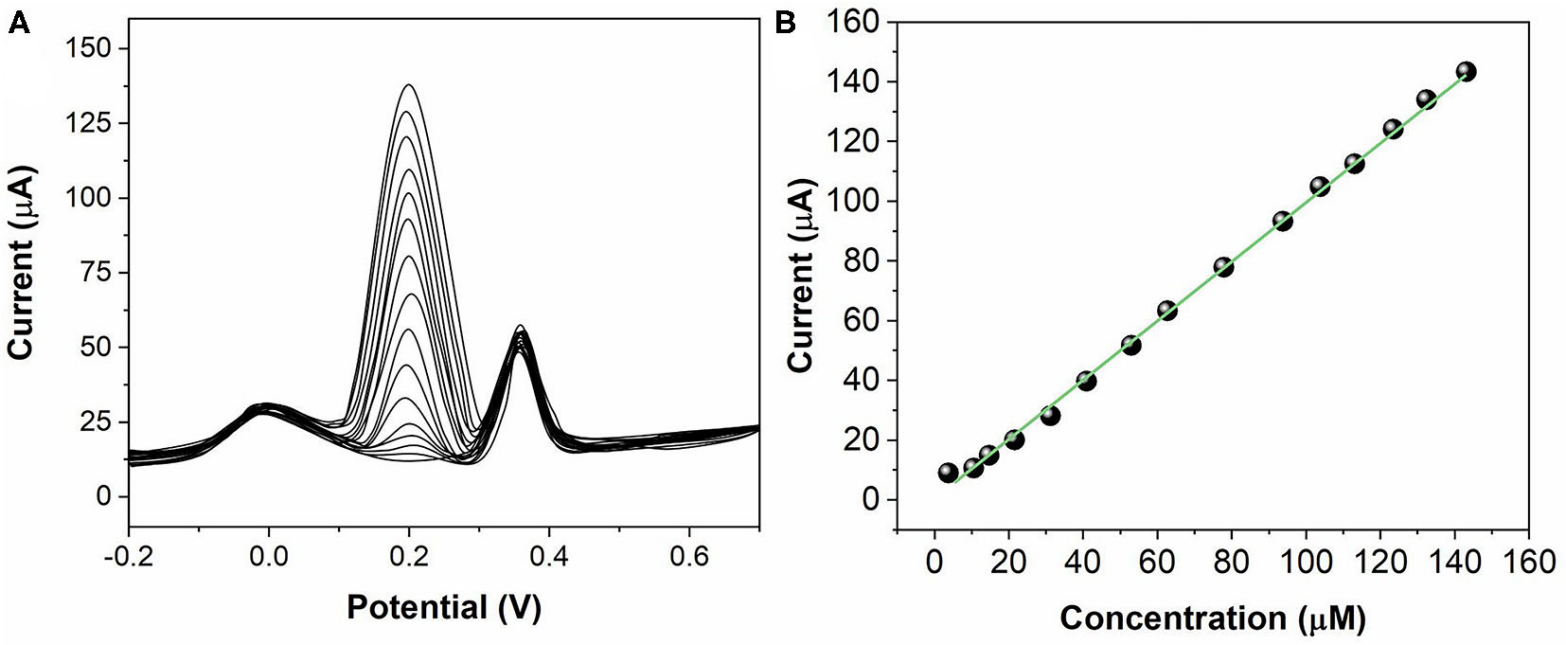
**(A)** SWVs for DA (2–150 μM) at ZnO NPs/GCE. **(B)** Calibration plots of DA concentration vs. current.

**Figure 7:**
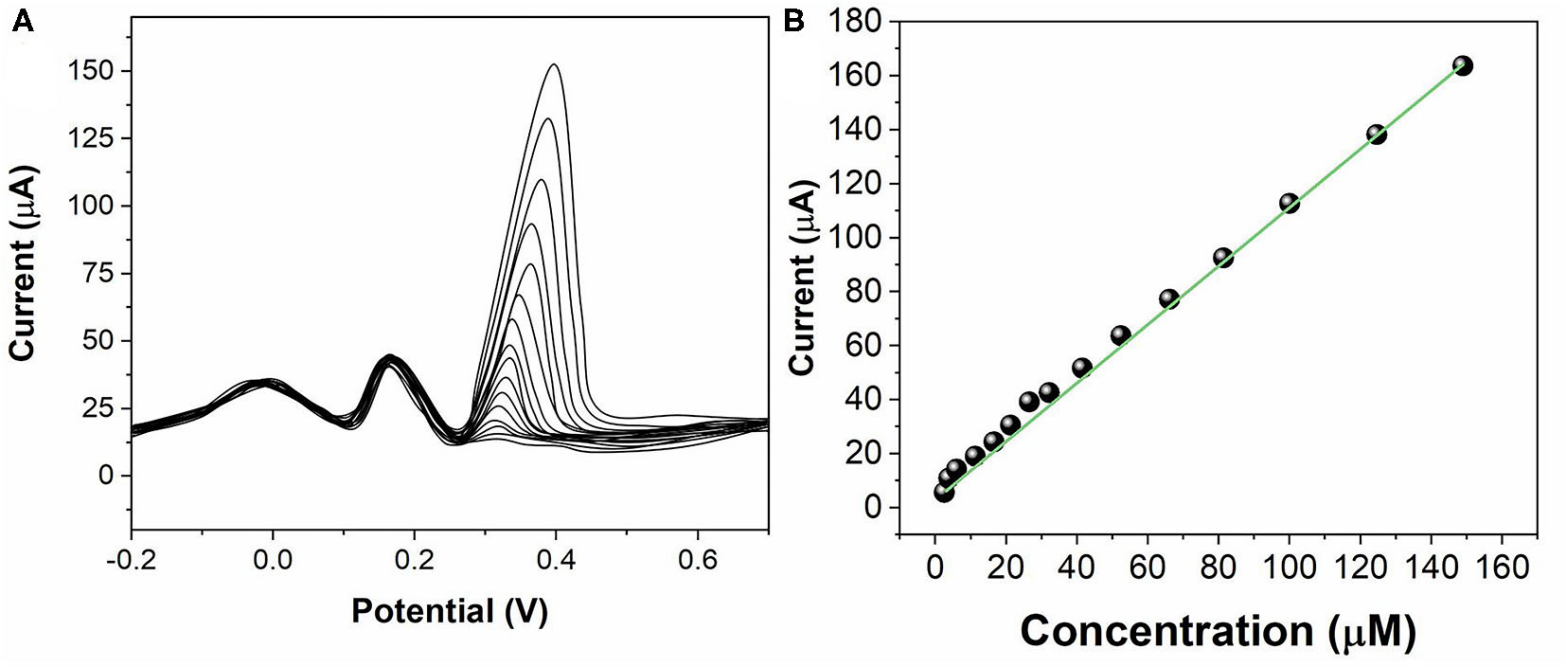
**(A)** SWVs for UA (0.2–150 μM) at ZnO NPs/GCE. **(B)** Calibration plots of UA concentration vs. current.

The sensing activity of ZnO NPs/GCE has been tested for individual AA, DA, and UA. During the test, the concentration of one analyte was changed, whereas the other two analytes remained the same. Figure 5A shows the SWV profiles of the ZnO NPs/GCE toward AA in the concentrations range between 50 and 1,000 μM. The peak currents exhibited a linear regression from 50 to 1,000 μM (Figure 5B) with an equation of *I*_pa(AA)_ = 5.3662 + 0.10771 C_AA_ (*R*^2^ = 0.9976). Figure 6A shows the SWV profiles of the ZnO NPs/GCE toward DA in the concentrations range between 2 and 150 μM. The peak currents exhibited a linear regression from 2 to 150 μM (Figure 6B) with an equation of *I*_pa(DA)_ = 0.6997 + 1.02465 C_AA_ (*R*^2^ = 0.9985). Figure 7A shows the SWV profiles of the ZnO NPs/GCE toward UA in the concentration range between 0.2 and 150 μM. The peak currents exhibited a linear regression from 0.2 to 150 μM (Figure 7B) with an equation of *I*_pa(DA)_ = 7.7951 + 1.07205 C_AA_ (*R*^2^ = 0.9977). The limits of detections of AA, DA, and UA using ZnO NPs/GCE were calculated to be 18.4, 0.75, and 0.11 μM, respectively.

**Figure 8:**
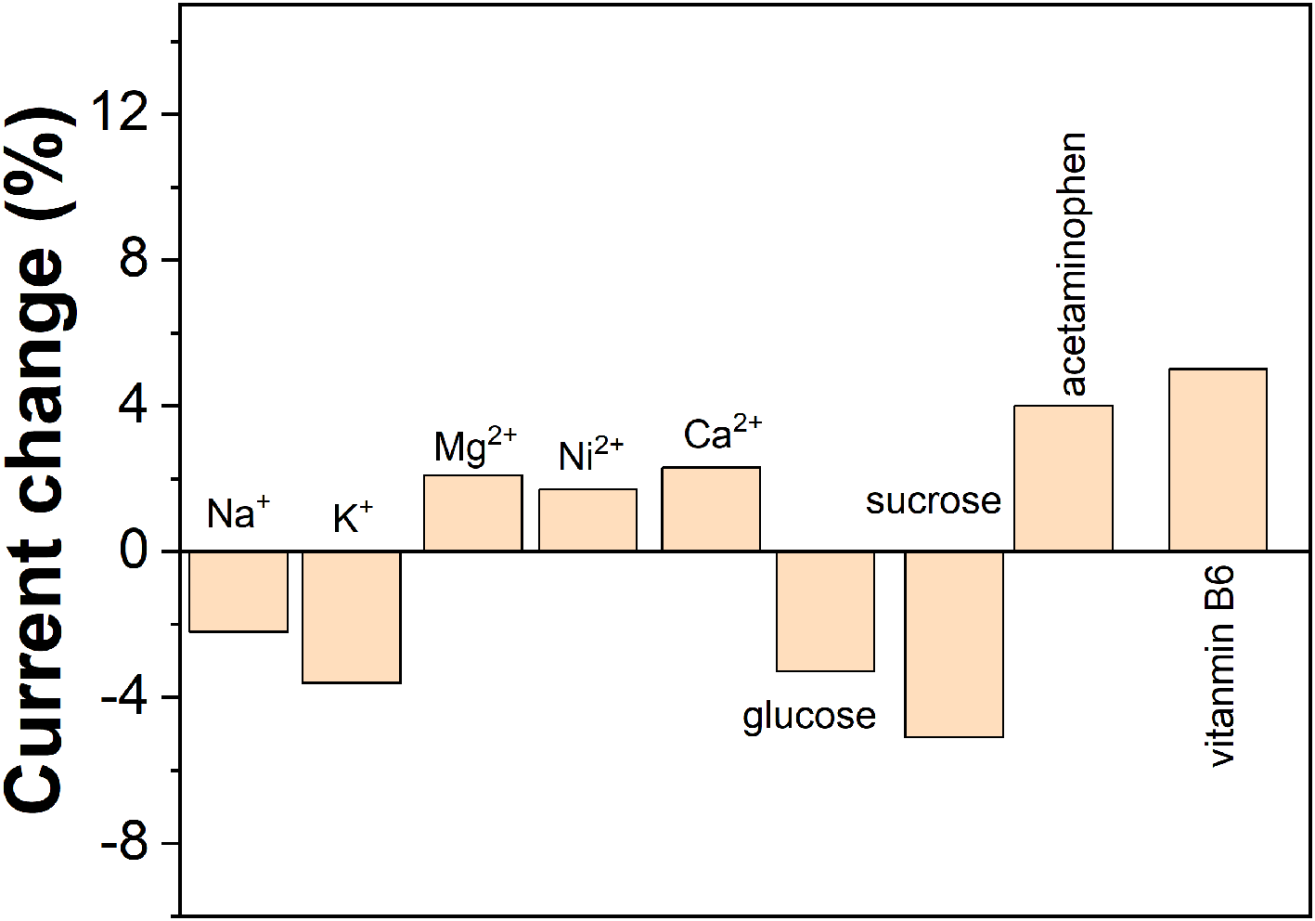
Anti-interference property of the ZnO NPs/GCE.

The stability of the ZnO NPs/GCE was tested by 10 successive measurements in three analytes. The responses remained almost stable with relative standard deviation (RSD) of 3.2, 3.7, and 4.5% for AA, DA, and UA, respectively. The reproducibility of the ZnO NPs/GCE was investigated by six individual ZnO NPs/GCE toward three analytes. The RSD was calculated to be 2.6, 3.1, and 4.4% for AA, DA, and UA, respectively. The selectivity of the ZnO NPs/GCE was tested by the presence of several potential interferences. As shown in Figure 8, 50-folds of common ions such as Na^+^, K^+^, Mg^2+^, Ni^2+^, and Ca^2+^ and 20-folds of glucose, sucrose, vitamin B_6_, and acetaminophen exhibited no interference on the sensing.

To illustrate the applicability of the ZnO NPs/GCE for real sample analysis, measurements were carried out in vitamin C (labeled as 50 mg/mL) and DA hydrochloride tablet (labeled as 20 mg/mL) by employing the standard addition method. As shown in Supplementary Table 1, the recovery of the spiked samples ranged between 93.49 and 102.01%, indicating the successful application of the ZnO NPs/GCE for the determination of AA, DA, and UA in real samples.

## Conclusion

We proposed an electrochemical sensor based on ZnO NPs for the simultaneous determination of AA, DA, and UA. The sensor showed a stronger ability to oxidize AA, DA, and UA compared with that of a bare GCE. The ZnO NPs/GCE exhibited a linear regression from 50 to 1,000 μM for AA, a linear regression from 2 to 150 μM for DA and a linear regression from 0.2 to 150 μM for UA. The limit of detections of AA, DA, and UA using ZnO NPs/GCE were calculated to be 18.4, 0.75, and 0.11 μM, respectively. The results suggest that ZnO NPs can be considered as an excellent candidate for electrochemical sensing.

## Data Availability Statement

The original contributions presented in the study are included in the article/Supplementary Material, further inquiries can be directed to the corresponding author/s.

## Author Contributions

YP and ZH contributed conception and design of the study. JZ and YP conducted electrochemical experiments. YH and CH performed the statistical analysis. YP and ZH wrote the manuscript. All authors contributed to manuscript revision, read, and approved the submitted version.

## Conflict of Interest

YP was employed by company Shanghai Electric Group Co., Ltd. and Zhenjiang Hongxiang Automation Technology Co., Ltd. CH was employed by company Beijing Kanghong Biomedical Co., Ltd. The remaining authors declare that the research was conducted in the absence of any commercial or financial relationships that could be construed as a potential conflict of interest.
