# Supplementary material for: Preparation of Electrochemical Sensor Based on Zinc Oxide Nanoparticles for Simultaneous Determination of AA, DA, and UA
Source: Front Chem. 2020 Nov 25;8:592538. doi: 10.3389/fchem.2020.592538 (PMC7723903; doi:10.3389/fchem.2020.592538)
Supplement: Supplementary file 1 [file Table_1.DOCX]

**Supplemental Information**

**Table S1.** Results for AA, DA and UA determination in Vitamin C tablet and DA hydrochloride table obtained by the G-30 under the optimum conditions.

| Sample |  | Found (μM) | Spiked (μM) | Found (μM) | Recovery (%) |
| --- | --- | --- | --- | --- | --- |
| Vitamin C tablet | AA | 47.62 | 100 | 149.52 | 99.68 |
|  | DA | 0 | 30 | 30.60 | 102.00 |
|  | UA | 0 | 30 | 28.95 | 96.50 |
| DA hydrochloride tablet | AA | 0 | 100 | 102.01 | 102.01 |
|  | DA | 0 | 30 | 29.98 | 99.93 |
|  | UA | 20.71 | 30 | 47.41 | 93.49 |
